# Trace and Heavy Metals in Locally and Imported Spices Sold on Markets in Accra Metropolis, Ghana

**DOI:** 10.1155/2024/3168279

**Published:** 2024-10-24

**Authors:** Isaac Osei-Safo, Kodwo Miezah, Lyndon Nii Adjiri Sackey, Junias Adusei-Gyamfi, Ayamba Malik Abdul

**Affiliations:** ^1^Ghana Standards Authority, Analytical Laboratory, Accra, Ghana; ^2^Department of Environmental Science, Faculty of Biosciences, College of Science, Kwame Nkrumah University of Science and Technology, Kumasi, Ghana

## Abstract

Spices enhance food's colour, aroma and palatability. The main objective of this study was to assess the levels of heavy metals in the most common spices used in Ghanaian and worldwide cuisines. Ninety samples were obtained directly from local marketplaces in the Accra Metropolis (Madina, Kaneshie and Makola). After microwave digestion, the samples' levels of arsenic, iron, lead, cadmium and zinc were measured using an inductively coupled plasma-mass spectrometer (ICP-MS). Iron, zinc, arsenic, cadmium and lead levels in specified natural spices varied from 0.022 mg/kg to 5.814 mg/kg, 0.056 mg/kg to 0.895 mg/kg, not detected to 14.012 mg/kg, 0.02 mg/kg to 0.45 mg/kg and not detected to 3.583 mg/kg, respectively. The toxic metals arsenic and lead in turmeric powder, whole rosemary and garlic, as well as lead in ginger, were slightly above the Codex, but below the FAO/WHO permissible level. All spices in this study had THQ and HI values of less than one, indicating that consumers will experience no potential health hazards from consuming specific metals through spices. However, continual scrutiny should be maintained over time due to bioaccumulation in humans.

## 1. Introduction

Spices are the moist or dry portions of plants that have long been used to improve food and beverages' colour, scent and taste. The bark, buds, bulb, leaves, fruit, seeds, rhizome or roots of a plant can contribute to the acceptability of foods such as soups, stir-fries and pasta meals [1]. Most of these are fragrant, aromatic and pungent. Metals are naturally occurring elements in the Earth's crust. They get into our bodies through food, water and air. Metals with more than 5 g/mL density are considered heavy metals. Environmental contamination by these metals has been a growing environmental and global public health problem in recent years. Furthermore, human exposure has significantly increased due to widespread industrial and agricultural use [2, 3]. Heavy metal poisoning in the food chain is mostly caused by air pollution from automotive exhaust, agricultural waste such as fertilizers, pesticides leaking into water bodies, pharmaceutical waste, home and industrial waste and smelting and process wastes from mining and other industries [4–7].

Most heavy metals are nonbiodegradable, and their bioaccumulation is due to their biological availability and extended biological half-life. Food is the primary nonoccupational source of heavy metal exposure for humans [8, 9]. Foods of various types, including spices, when exposed to heavy metals in many ways, lead to health hazards when consumed by humans. The trace metals found in spices and medicinal herbs are vital for the functioning of some cells in humans. A variety of these metals in the form of trace elements are essential for many functions in the human body, and their deficiency leads to severe symptoms. However, high levels of these metals become hazardous because of their deleterious effects on human health. This is imperative that we understand the level of these metals in our food [10]. Many health problems have been linked to man's exposure to harmful heavy metals such as cadmium, lead, copper and zinc [11]. When ingested over a certain threshold, lead (Pb) can raise blood pressure and negatively affect key organs such as the kidneys and the brain. Cadmium (Cd) poisoning has been related to several respiratory ailments, renal failure and cardiovascular problems. Despite being a vital mineral, zinc (Zn) overdose can cause fever, nausea and general weakness. Although a lack of iron causes anaemia, too much iron in children can cause gastrointestinal and skin problems [12, 13]. In general terms, Cd, Pb, Cr, Cu, Zn, As and Ni are the most hazardous heavy metals in the environment [14]. Heavy metals are deleterious because they tend to bioaccumulate and are taken up and stored faster than degraded or excreted [15].

With a rising understanding of the importance of spices in human diets and an increase in their use, it is vital to monitor various contaminants such as heavy metals to minimize health hazards [16]. Natural food spices such as pepper, garlic and mustard have been reported to contain substantial quantities of some trace metals [17]. Lead, despite being the most ecotoxic metal, naturally occurs in plants. Recently, Pb and Cd were listed by the Agency for Toxic Substances and Disease Registry [18] as second and seventh-priority toxic substances, respectively. Consequently, monitoring the hazardous effects of the heavy metals in common spices is imperative, since many spices have also been shown to have antidiabetic, anti-inflammatory and antihypertensive properties [19]. However, there is little information available about the safety of these spices, both imported and locally produced with respect to heavy metal contamination in Ghana.

In Ghana, the increasing prevalence of anthropogenic activities, such as intensive mining, irrigation with contaminated water and the application of pesticides and fertilizers, has heightened the risk of heavy metal contamination in spices [20]. The growing consumption of spices in Ghana and globally necessitates an investigation into their heavy metal content to mitigate potential contamination risks. Monitoring these levels is crucial for assessing the health impacts of spice consumption and providing essential data on spice safety in the country. This study aims to evaluate the content of selected heavy metals (As, Cd, Zn, Fe and Pb) in common spices produced locally and imported into markets within the Accra Metropolis of Ghana.

## 2. Materials and Methods

### 2.1. Study Area

**Figure 1:**
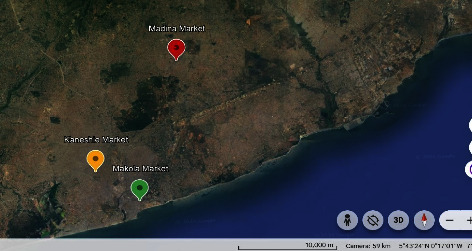
Map of Accra Metropolis, Greater Accra Region—Google 2023.

The study areas of this project are the markets in Madina, Kaneshie and Makola, all in the Greater Accra region of Ghana. The above markets were selected since they are the main distribution points for natural spices within the metropolis. Accra Metropolitan Area consists of many submetropolitan areas, which include Kpeshie, Osu-Clottey, Central Ayawaso, East Ayawaso, West Ayawaso, Ashiedu-Keteke, Okai-koi North, Okai-koi South, Ablekuma South and Ablekuma North. Accra covers a land mass of about 1,261 km^2^ and lies geographically within longitude 0° 03′ and 0° 25′ West and latitude 5° 30′ and 5° 53′ North (Figure 1) with the following link https://earth.google.com/earth/d/1dIPIzqaMEJF-cpUmKzCBiD9E1Y3NiT6J?usp=sharing.

### 2.2. Sampling Collection and Preparation of Spices for Analysis

Table 1 listed raw and processed spice samples that were randomly purchased from three sellers within a market thus, ten spice samples were sampled and a total of ninety samples were sampled from the three markets. The samples were sent to the laboratory using polyethylene bags in an ice chest, prepared and kept in the freezer until it was analysed.

The whole samples were minced by a laboratory blender, and homogenized and powdered samples were only homogenized according to the sampling collection from each seller. The samples were stored in a conditioned cup in the refrigerator before analysis.

### 2.3. Chemicals, Reagents and Apparatus

Nitric acid (65–69%), Trace Metal Grade (P/N A509-P212), and hydrogen peroxide (30–32%), Trace Metal Grade (P/N H/1820/15) were procured from Thermos Fisher Scientific (Chicago, United States). Deionized water (18.20 MΩ·cm) was obtained from a PURELAB Classic water purification system in the metallic contaminants laboratory of GSA. Single-element standard solutions (As, Fe, Zn, Pb and Cd), each at 1000 mg/L, were purchased from Inorganic Ventures (Christiansburg, Virginia, USA). Different analytical procedures have been reported in the literature for determining trace elements and heavy metals in spices. The inductively coupled plasma-mass spectrometry (ICP-MS) is commonly accepted as the technique of choice and offers the advantage of high sensitivity, selectivity, wider dynamic range and robust performance. In addition to the analytical system of choice, sample preparation is also important. Microwave-assisted acid digestion is the most common and preferred sample preparation technique for analysis by ICP-MS due to its advantages over the open digestion method. When compared to the open digestion method, microwave digestion proficiently retains the volatile elements such as mercury in the sample solution, ensuring complete decomposition of the organic sample matrix at the same time [21].

### 2.4. Preparation of Spices for Metallic Analysis

One gram of prepared spice was weighed into a Teflon container, and 5 mL of nitric acid (HNO_3_) and 3 mL of hydrogen peroxide (H_2_O_2_) were added. The Teflon containers were then put into their shields tightly covered and later arranged into the microwave digester for the wet digestion process. The digester was operated at a temperature of 170°C and a pressure of 50 bar for 45 mins for complete digestion of samples. It was allowed to cool for 30 minutes, and the digested samples were then poured into 50-mL centrifuge tubes and topped up to the 25-mL mark with deionized water for quantification. A certified reference material (CRM; FAPAS 07340) was added to each batch of samples. This was done to ensure the quality assurance of the analytical work of the study. Each batch also had a sample blank prepared just like the sample.

### 2.5. Working Standards Preparation

Intermediate mixed working standard As, Fe, Zn, Pb and Cd solutions were prepared from their respective stock standard solutions of 1000 mg/L (ppm) by serial dilution. 10,000 µg/L intermediate bulk standard solution was prepared by pipetting 1 mL of each of the stock standard solutions into a 100-mL volumetric flask and topping it up to the 100-mL mark with 2% nitric acid solution. Another 100 µg/L intermediate standard solution was prepared from the 10000 µg/L by pipetting and diluting 1 mL with 2% nitric acid in a 100-mL volumetric flask to the mark. The working standard solutions of 1 µg/L, 5 µg/L, 10 µg/L, 20 µg/L and 50 µg/L were prepared by diluting 0.5 mL, 2.5 mL, 5.0 mL, 10 mL and 25 mL of the 100 µg/L mixed standard solution of As, Fe, Zn, Pb and Cd with 2% nitric acid in 50-mL volumetric flasks as shown in Table 2.

The 2% nitric acid solution was prepared by measuring 20 mL of concentrated nitric acid into a 1L volumetric flask containing distilled water and making it up to the mark with deionized water.

### 2.6. Elemental Analysis

Agilent 7700 Series ICP-MS manufactured in California, USA, was used to analyse As, Fe, Zn, Pb and Cd in the various spices (Table 3) [22, 23].

### 2.7. Quality Assurance

The accuracy of the analytical method was evaluated by analysing a CRM (Cocoa Powder, FAPAS 07340) (Table 4) with the prepared samples. A 0.5 g of the CRM was weighed into the digestion vessel with the samples (Table 1) and taken through the same digestion procedure as the spice's samples. The CRM and the sample digests were analysed for As, Fe, Zn, Pb and Cd. The percentage recovery of the CRM and the coefficient of regression of the calibration curves of the study indicate good analytical work done (Appendices 2Ato 2E). The percentage recovery of the CRM and the coefficient of regression of the calibration curves of the study indicate good analytical work done.

The limit of detection (LOD) and limit of quantification (LOQ) of As, Fe, Zn, Pb and Cd for the Agilent 7700 Series ICP-MS are shown in Table 5.

### 2.8. Health Risk Assessment of Spices Consumption

The estimated daily intake (EDI), target hazard quotient (THQ) and hazard index (HI) were used to evaluate the possible health concerns associated with ingesting heavy metals through spices. The daily amount taken, body weight and element concentrations in spices define the EDI value as shown in the following equation:

(1) $$\begin{array}{c} \text{EDI}=\frac{\text{concentration of metal}\times \text{ingestion rate}}{\text{average body weight}\times 1000}, \end{array}$$

where EDI is estimated daily intake. There should be comma before the the concentration of heavy metal content in spices is mg/kg, ingestion rate gram/day person is 10 g/day/person and average body weight (kg) is 60 kg.

THQ is used to assess the noncarcinogenic risks of long-term exposure to contaminants in Spices, and the calculations were done using the following equation:

(2) $$\begin{array}{c} \text{THQ}=\frac{\text{EDI}}{\text{RfD}}, \end{array}$$

where reference dose (RfD) values for each metal of interest are Cd (0.001), As (0.003), Pb (0.004), Zn (0.3) and Fe (0.7) mg/kg per day, respectively [24].

To calculate the overall noncarcinogenic risk to human health posed by exposure to multiple pollutants, the HI was developed. HI is the sum of the HQ for all heavy metals found in spices, as shown in the following equation:

(3) $$\begin{array}{c} \text{HI}=\sum \text{THQ}. \end{array}$$

If the values of THQ/HI ≥1 imply that the population may have negative health effects, while if THQ/HI <1, the population is not expected to encounter any obvious negative consequences [25].

### 2.9. Statistical Analysis

GenStat 12th Edition was used to analyse the data, and Microsoft Excel, 2016, was used for graphical representations.

## 3. Results

The contents of most heavy metals in spices in this study were lower than the [26, 27], Codex Alimentarius [28] and Commission Regulation (EU) 2015/1005 for heavy metals, except for Rosemary, Turmeric powder, and Garlic (Table 6). The levels of iron, zinc, arsenic, cadmium and lead in listed natural spices are presented in supplementary sheets ranging from 0.02 mg/kg to 5.81 mg/kg, 0.06 mg/kg to 0.90 mg/kg, not detected to 14.01 mg/kg, 0.02 mg/kg to 0.45 mg/kg and not detected to 3.58 mg/kg, respectively. Table 6 illustrates that the mean levels and standard deviation of iron, zinc, arsenic, cadmium, and lead in listed natural spices are presented in supplementary sheets ranging from 0.076 ± 0.042 to 4.086 ± 0.536 mg/kg, 0.096 ± 0.031 to 0.749 ± 0.106 mg/kg, 0.068 ± 0.052 to 9.831 ± 4.352 mg/kg, 0.051 ± 0.017 to 0.156 ± 0.054 mg/kg and 0.100 ± 0.110 to 2.016 ± 0.957 mg/kg, respectively.

## 4. Levels of the Heavy Metals in the Spices

### 4.1. Heavy Metals in Each Spice from the Three Markets

From Appendix 1B, an amount of 5.338 mg/kg, the highest concentration of Fe levels was found in white pepper from the Madina market, and the least concentration of about 0.072 mg/kg was also in garlic from the Kaneshie market. Ginger on the other hand contains the highest concentration of Pb from all the markets without any significant difference. Garlic recorded the highest concentration of As and Cd estimated as 13.620 mg/kg and 0.161 mg/kg all from the Madina market whereas green pepper and Nutmeg had the least concentration of As and Cd estimated as 0.115 mg/kg and 0.045 mg/kg from the Kaneshie and Madina markets, respectively. The highest concentration of Zn content was recorded in white pepper with an estimation of 0.808 mg/kg from the Makola market. However, the least Zn concentration was found in green pepper with an estimation of 0.093 mg/kg from the Kaneshie market.

### 4.2. Heavy Metals in Foreign and Local Spices

From Table 7, the foreign spices contain high concentration of As which are significantly different from the locals' ones. Pb and Fe contents were high in the local spices and significantly different from the foreign. Zn and Cd contents were not significantly different in the foreign and local spices. Hence, the foreign spices are less contaminated than the local spices.

### 4.3. Health Risk Assessment

Table 8 illustrates the toxic metal EDI values associated with each spice use. The analysis yielded the following EDI values for As, Fe, Pb, Cd and Zn: 0.0000113–0.0016385, 0.0000127–0.0006810, 0.0000652–0.0003360, 0.0000085–0.00026 and 0.0000160–0.0001248, respectively. The EDI value trends showed that As would lead to a daily increase in heavy metals consumed, with As > Fe > Pb > Cd > Zn. As and Cd levels in EDI values were usually the same except for rosemary and garlic spices. Given that all of the EDI values in this study fell below the RfD values for all components (Table 8), consuming the spices under study does not appear to represent a health concern except for the level of As in rosemary and garlic.

THQ values for all spices were less than one, suggesting no possible health risk from consuming spices. The study's hazardous metal THQ values for consuming spices were less than 1, as shown in Table 9. This result suggests that it is unlikely that using spices will result in the potentially harmful daily absorption of some hazardous metals. The combined noncarcinogenic effects of Pb, Fe, Zn, Cd and As can be expressed using the HI value. The HI values were less than 1 for each metal investigated by the ingestion of spices (Table 9).

## 5. Discussion

### 5.1. Levels of the Heavy Metals in the Spices

The highest concentration of Fe was in aniseed (4.086 mg/kg), and the least was in garlic (0.076 mg/kg). Ginger had the highest concentration of Pb (2.016 mg/kg), far more than twice the concentrations of the next highest aniseed (0.975 mg/kg). Garlic recorded the highest concentration of As and Cd, whereas green pepper and nutmeg had the least in As and Cd, respectively. The highest concentration of Zn content was in the white pepper. From the results, it can be ascertained that garlic, rosemary and turmeric powder contain high levels of As and Cd whereas aniseeds also contain high levels of Fe, all foreign or imported spices. However, the local spices had low levels of heavy metals. Also, there were high levels of Pb and Zn in the locally produced spices (ginger and white pepper), two of which were essential metals (Zn and Fe), although the foreign spices recorded relatively low levels. Compared to Codex Alimentarius [28] and Commission Regulation (EU) 2015/1005, levels of Cd in all the spices were generally low. Garlic had the highest concentration of As, followed by turmeric powder and rosemary. Concentrations of Fe in all the spices were above the Codex Alimentarius [28] and Commission Regulation (EU) 2015/1005. All the heavy metals assessed were within the acceptable limit except ginger, above the acceptable limit of 1.0 mg/kg. White pepper contained the highest concentration (0.749 mg/kg) of Zn, which is well below the Codex limit of 10 mg/kg. Ginger showed the highest concentration (2.016 mg/kg) of Pb, which is above the Codex limit of 1.0 mg/kg. According to [29], the results obtained showed that the concentrations of heavy metal in the korarima, red pepper, ginger and turmeric samples in mg/kg of dry weight were in the range of Fe (38.7–98.9), Mn (10.5–257), Zn (7.30–29.2), Cu (1.70–6.50), Cr (5.40–9.70), Cd (1.50–2.90), Pb (14.5–28.4) and Ni (3.90–6.70) which are by far higher in concentration than the results obtained from the study in the Accra Metropolis, Ghana. Islam et al., [30] stated that the highest concentrations of Pb (15.47 ± 1.93), Cd (1.65 ± 0.011), Cr (31.99 ± 3.97), Cu (18.84 ± 1.97) and Fe (9.29 ± 1.71) were observed in cardamom, coriander, bay leaf, cold water chili and black chili. Also, in comparison with [31] state, the following concentrations of Fe (32.22 ± 1.22–131.1 ± 3.26), As (ND to 0.12 ± 0.04), Cr (0.08 ± 0.01–3.2 ± 0.09), Pb (N.D–0.21 ± 0.02) and Cd (ND to 0.14 ± 0.08) in mg/kg were measured, and the As, Cd and Pb concentrations were relatively lower than the results obtained from the study in the Accra metropolis, Ghana.

### 5.2. Heavy Metals in Each Spice from the Three Markets

An amount of 5.338 mg/kg, the highest concentration of Fe levels was found in white pepper from the Madina market, and the lowest concentration of about 0.072 mg/kg also in Garlic from the Kaneshie market. Ginger, conversely, contains the highest concentration of Pb from all the markets without any significant difference. The results indicated that the Pb concentrations in Romania, Pakistan and Iraq ranged from 0.04 to 1.28 [32], 4.44 to 15.88 [33] and 3.21 to 6.98 [34] in mg/kg, respectively, were relatively higher when compared to current studies conducted with other countries. Pb is a hazardous heavy metal that binds to enzyme oxo-groups, interfering with haemoglobin formation and porphyrin metabolism nearly all the way through. Pb poisoning in humans has been connected to encephalopathy, seizures and mental retardation, among other health issues [35]. Pb is a cumulatively toxic substance that damages many human body systems, particularly in young children. Because it can accumulate and concentrate, it threatens the safety and health of living things, even at very low concentrations, which are considered extremely dangerous [27]. Garlic recorded the highest concentration of As and Cd estimated as 13.620 mg/kg and 0.161 mg/kg, all from the Madina market, whereas green pepper and nutmeg had the lowest concentration of As and Cd estimated at 0.115 mg/kg and 0.045 mg/kg from the Kaneshie and Madina markets, respectively. The presented study was compared with a study from Poland, and their result revealed that Cd was low and varied from <LOQ (0.020 mg/kg) to 0.082 mg/kg [36]. None of their analysed samples contained Cd levels exceeding the permitted limits for that metal. The results obtained in this study and the results published in the literature are similar levels of Cd in samples of herbs and spices analysed. The highest concentration of Zn content was recorded in white pepper, with an estimation of 0.808 mg/kg from the Makola market. However, the lowest Zn concentration was found in green pepper, with an estimated 0.093 mg/kg from the Kaneshie market, considering that all the samples had low Fe levels. Every result was under the 300 mg/kg WHO allowable range. The results of this study and a study on spices conducted in Tikrit and Baghdad city are comparable. We discovered that the iron concentration ranged between 6.870–19.370 mg/kg [37] and 32–490 mg/kg [38].

### 5.3. Heavy Metals in Foreign and Local Spices

The foreign spices contain high concentrations of the As, significantly different from the local ones. It is estimated that several million people are exposed to arsenic chronically throughout the world, especially in countries such as Bangladesh, India, Chile, Uruguay, Mexico and Taiwan, where the groundwater is contaminated with high concentrations of arsenic [39]. Natural levels of arsenic in soil usually range from 1 to 40 mg/kg, but pesticide application or waste disposal can produce much higher values [40]. Arsenic exposure affects virtually all organ systems, including the cardiovascular, dermatologic, nervous, hepatobiliary, renal, gastrointestinal and respiratory systems [41]. Research has also pointed to significantly higher standardized mortality rates for cancers of the bladder, kidney, skin and liver in many areas of arsenic pollution [42]. The severity of adverse health effects is related to the chemical form of arsenic and is also time- and dose-dependent [43]. The Pb and Fe contents were high in the local spices and significantly different from the foreign ones. In some sub-Sahara African countries, ground and some surface waters are mostly used to irrigate small plants such as spices and herbs. There have been high levels of heavy metals in groundwater, and few surface water exceeds the permissible limit of water. Destructive mining into surface water also contributes to the high levels of heavy metals [44]. In Ghana, the increasing prevalence of anthropogenic activities, such as intensive mining, irrigation with contaminated water, and the application of pesticides and fertilizers, has heightened the risk of heavy metal contamination in spices [20]. The ionic mechanism of lead toxicity occurs principally with the ability of lead metal ions to replace other bivalent cations (Ca^2+^, Mg^2+^ and Fe^2+^) and monovalent cations (Na^+^), which intern ultimately disturbs the biological metabolism of the living cells [45]. Ionic lead toxicity may also substitute calcium even in picomolar concentration, which affects the protein kinase C, which intern influence neural excitation and memory storage [46]. Hence, the consumption of local spices of this nature could cause harm to human health over time. The presence of these heavy metals in food as a habit may result in the accumulation of these metals in human organs. The accumulation of heavy metals can have middle-term and long-term health risks [47]. The exposure of humans to these metals, even in small proportions for a long time, leads to kidney problems, lung diseases, osteoporosis, high blood pressure problems in liver function and the destruction of brain cells. Additionally, it works to break down fatty acids and is considered a carcinogen, and the increase in its concentration in food causes harm to the health of the consumer [37]. Zn and Cd contents were not significantly different in the foreign and local spices. Hence, the foreign spices are less contaminated than the local spices.

### 5.4. Health Risk Analysis

According to Islam et al., [30], approximately 37% of Cr and 5% of Fe EDI were above the RfD. All spices had THQ values below acceptable, while 37% of them had Total Target Hazard Quotient (TTHQ) values above average, suggesting adverse health consequences for consumers. The obtained results are not in conformity with data from similar studies conducted by the authors of [48–50] on the amount of metal contamination in fruits where HI values were higher. According to [51], the high HI values observed suggest that the longer use of the fruit product might present a possible noncarcinogenic health risk to the consumers even at a lower concentration. Much research has been done on levels of heavy metal contamination of functional foods, spices medicinal herbs and herbal preparations in various regions of Ghana [20, 52, 53]. It was discovered that the mean THQ value for Pb was less than that of Pakistan (0.281) and Nigeria (0.125) [33, 54]. Considering every metal, the total THQ values for the investigated spices varied from 0.135 to 8.328. Certain spices had been considered the possible health risk. The following foods have extremely high total THQ values of Cr: green chili, ginger, coriander leaf and all varieties of turmeric and chili powder. Compared to earlier studies carried out in Poland, Pakistan, Egypt and Nigeria, our research had a lower total THQ value [33, 54, 55].

Due to the high risk associated with human consumption of contaminated foods and herbs, an effective continuous monitoring programme must be developed. This suggests that intake of the spices may have negligible effects on consumers' health. However, the high levels of toxic metals (As and Pb) may suggest the need for regular monitoring of the spices for contamination. Also, monitoring and sensitization of farmers on the effects of indiscriminate and excessive use of agrochemicals, industrialisation, rapid urbanization and unregulated mineral extraction activities are very necessary to protect the health of consumers and preserve soil quality.

## 6. Conclusions and Future Perspective

The study showed that iron, zinc and cadmium were present in all seasonings, whereas 95% of the spices contained lead and arsenic. The highest concentration of Fe was in aniseed, and the least was in garlic. Ginger had the highest concentration of Pb, far more than twice the concentrations of aniseed and Negro pepper. Garlic recorded the highest concentration of As and Cd, whereas green pepper and Nutmeg had the least in As and Cd, respectively. The highest concentration of Zn content was in the white pepper. The levels of toxic metals arsenic (As) in turmeric powder, rosemary, and garlic and lead (Pb) in ginger were slightly above the Codex Alimentarius [28] and Commission Regulation (EU) 2015/1005 for heavy metals as regards maximum levels of lead in certain foodstuffs. Some foreign spices such as garlic, rosemary, aniseeds and turmeric powder, and some local spices such as Ginger and Negro pepper are dangerous to consume due to the high levels of the following heavy metals; Pb, Cd and As, which tends to disrupt metabolism and growth in the human system. It is advised that the intake rate of the above spices should be reduced to minimize its effect on humans. Further studies should consider specific sources (e.g., soil and water sources of irrigation) of contamination by heavy metals in the natural spices and the health impact on the consumption. Due to the varying sources of contamination by heavy metals in food and the large number of spices in our markets, the regulating body, Ghana Food and Drugs Authority (FDA-Ghana), should regularly monitor heavy metals [56].

## Figures and Tables

**Table 1:** Sample code, spice and country of origin.

Sample Code	Sample (spice)	Origin
S1, S2 and S3	Ginger—Rhizome	Ghana (M1)
S1, S2 and S3		Ghana (M2)
S1, S2 and S3		Ghana (M3)

S1, S2 and S3	Garlic—bulb	India (M1)
S1, S2 and S3		India (M2)
S1, S2 and S3		Morocco (M3)

S1, S2 and S3	Rosemary—leaves	China (M1)
S1, S2 and S3		China (M2)
S1, S2 and S3		China (M3)

S1, S2 and S3	Aniseed (Nketekete)—seeds	Mali (M1)
S1, S2 and S3		Mali (M2)
S1, S2 and S3		Mali (M3)

S1, S2 and S3	Negro pepper (Hhwintia)—fruit pods	Ghana (M1)
S1, S2 and S3		Ghana (M2)
S1, S2 and S3		Ghana (M3)

S1, S2 and S3	Nutmeg—fruit pods	France (M1)
S1, S2 and S3		France (M2)
S1, S2 and S3		Morocco (M3)

S1, S2 and S3	Green pepper (mako hwam)—fruit pods	Ghana (M1)
S1, S2 and S3		Ghana (M2)
S1, S2 and S3		Ghana (M3)

S1, S2 and S3	White pepper (Famu Wisa)—fruit pods	Ghana (M1)
S1, S2 and S3		Ghana (M2)
S1, S2 and S3		Ghana (M3)

S1, S2 and S3	Tumeric powder∗—rhizome	India (M1)
S1, S2 and S3		India (M2)
S1, S2 and S3		India (M3)

S1, S2 and S3	Black pepper (Soro wisa)—fruit pods	Malaysia (M1)
S1, S2 and S3		Malaysia (M2)
S1, S2 and S3		Malaysia (M3)

*Note:* Madina—M1, Kaneshie—M2, Makola—M3.

^∗^Processed spice.

**Table 2:** The preparation of working standards for the calibration curve.

Concentration of stock solution (ppb)	Volume of aliquot taken from the stock solution to prepare the working standards (mL)	Volume of the working standards prepared (mL)	Concentration of working standards prepared (ppb)
1000000	1	100	10000
10000	1	100	100
10000	0.5	50	1
10000	2.5	50	5
10000	5.0	50	10
10000	10	50	20
10000	25	50	50

**Table 3:** The operational parameters for ICP-MS.

Metre	Analysis mode	Unit
Plasma gas	15	L/min
Auxiliary gas	0–1.0	L/min
Helium gas	3–5 and 10–12	mL/min
Forward power	700 to 1600	W
Ar gas tank pressure	500–700	kPa
Inlet and exhaust temperature	15–45 and 45–55 rpt	°C
Carrier gas	0.8–1.3	L/min
Reflected power	<20	W
Plasma frequency	26.0–28.0	MHz
Detector	Single mass spectroscopy	

**Table 4:** The percentage recovery of CRM.

Analyte in certified reference material (CRM) (FAPAS 07340)	Assigned values of analyte in CRM (ppm)	Results obtained from the analysis of the CRM (ppm)	Percentage recovery for the analysis of CRM (%)
Pb	0.200 ± 0.041	0.206	103
As	0.342 ± 0.062	0.370	108
Fe	1.431 ± 0.089	1.428	99.8
Zn	0.070 ± 0.006	0.071	101.4
Cd	0.299 ± 0.018	0.2785	93.14

**Table 5:** The limit of detection (LOD) and limit of quantification (LOQ).

Analyte	LOD (mg/l)	LOQ (mg/l)
Pb	0.003	0.010
Cr	0.003	0.0
Fe	0.007	0.020
As	0.017	0.050
Zn	0.007	0.020

**Table 6:** Estimated mean concentrations of the heavy metals in the spices from the markets.

Spice	As (mg/kg)	Zn (mg/kg)	Cd (mg/kg)	Fe (mg/kg)	Pb (mg/kg)
Green pepper	0.068 ± 0.052^a^	0.096 ± 0.031^a^	0.084 ± 0.035^ab^	1.985 ± 0.892^d^	0.100 ± 0.110^a^
Black pepper	0.075 ± 0.032^a^	0.416 ± 0.238^bc^	0.097 ± 0.052^abc^	1.786 ± 1.015^cd^	0.714 ± 0.321^cd^
Ginger	0.083 ± 0.019^a^	0.654 ± 0.043^de^	0.088 ± 0.030^abc^	1.310 ± 0.353^cd^	2.016 ± 0.957^e^
White pepper	0.126 ± 0.011^a^	0.749 ± 0.106^e^	0.138 ± 0.045^cd^	3.465 ± 1.401^e^	0.905 ± 0.074^d^
Nutmeg	0.156 ± 0.088^a^	0.306 ± 0.135^b^	0.051 ± 0.017^a^	1.158 ± 0.21^bc^	0.391 ± 0.230^ab^
Negro pepper	0.168 ± 0.029^a^	0.542 ± 0.198^cd^	0.103 ± 0.034^abcd^	1.928 ± 1.323^cd^	0.973 ± 0.084^d^
Aniseed	0.204 ± 0.027^a^	0.363 ± 0.077^b^	0.125 ± 0.012^bcd^	4.086 ± 0.536^e^	0.975 ± 0.185^d^
Rosemary	4.726 ± 2.780^b^	0.699 ± 0.101^e^	0.074 ± 0.035^ab^	0.397 ± 0.131^ab^	0.741 ± 0.177^cd^
Turmeric powder	4.973 ± 1.610^b^	0.351 ± 0.169^b^	0.120 ± 0.060^bcd^	1.292 ± 0.052^cd^	0.581 ± 0.130^bc^
Garlic	9.831 ± 4.352^c^	0.647 ± 0.143^de^	0.156 ± 0.054^d^	0.076 ± 0.042^a^	0.780 ± 0.103^cd^
LSD	1.687	0.130	0.054	0.795	0.319
CV (%)	21.4	13.1	2.1	4.4	20.4
Codex limit	4.0	10.0	0.1	N/A	1.0
WHO limit	5	50	0.2	300	10

*Note:* Values are means from three replicate analyses. Those with the same superscripts in the same column are not significantly different at *p* > 0.05.

**Table 7:** Estimated mean concentrations of the heavy metals in foreign and local spices.

	Fe (mg/kg)	As (mg/kg)	Zn (mg/kg)	Cd (mg/kg)	Pb (mg/kg)
Foreign spices	1.466^a^	3.328^b^	0.464^a^	0.104^a^	0.697^a^
Local spices	2.172^b^	0.111^a^	0.510^a^	0.103^a^	1.010^b^
LSD	0.50	2.27	0.03	0.00	0.22
CV (%)	27.5	132.3	6.8	0.5	25.9

*Note:* Values are means of replicate analysis in Table. Those with the same superscripts in the same column are not significantly different at *p* > 0.05.

**Table 8:** Estimated daily intake of the heavy metals analysed.

Spice	As (mg/kg)	Zn (mg/kg)	Cd (mg/kg)	Fe (mg/kg)	Pb (mg/kg)
Green pepper	1.13 × 10^−5^	1.60 × 10^−5^	1.40 × 10^−5^	33.08 × 10^−5^	16.67 × 10^−5^
Black pepper	1.25 × 10^−5^	6.93 × 10^−5^	1.62 × 10^−5^	29.97 × 10^−5^	11.90 × 10^−5^
Ginger	1.38 × 10^−5^	10.90 × 10^−5^	1.47 × 10^−5^	21.83 × 10^−5^	33.60 × 10^−5^
White pepper	2.10 × 10^−5^	12.48 × 10^−5^	2.30 × 10^−5^	57.75 × 10^−5^	15.08 × 10^−5^
Nutmeg	2.60 × 10^−5^	5.10 × 10^−5^	0.85 × 10^−5^	19.30 × 10^−5^	6.52 × 10^−5^
Negro pepper	2.80 × 10^−5^	9.03 × 10^−5^	1.72 × 10^−5^	32.13 × 10^−5^	16.22 × 10^−5^
Aniseed	3.40 × 10^−5^	6.05 × 10^−5^	2.08 × 10^−5^	68.10 × 10^−5^	16.25 × 10^−5^
Rosemary	78.80 × 10^−5^	11.65 × 10^−5^	1.23 × 10^−5^	6.62 × 10^−5^	12.35 × 10^−5^
Turmeric powder	2.67 × 10^−5^	5.85 × 10^−5^	2.00 × 10^−5^	21.53 × 10^−5^	9.63 × 10^−5^
Garlic	163.85 × 10^−5^	10.78 × 10^−5^	26.00 × 10^−5^	1.27 × 10^−5^	13.00 × 10^−5^
RfD [24]	0.03	0.3	0.001	0.7	0.004

**Table 9:** Target hazard quotient and hazard index.

Spice	As (mg/kg)	Zn (mg/kg)	Cd (mg/kg)	Fe (mg/kg)	Pb (mg/kg)	Hazard index (HI)
Green pepper	3.76 × 10^−3^	5.33 × 10^−5^	1.40 × 10^−2^	4.72 × 10^−4^	4.17 × 10^−2^	0.07
Black pepper	4.17 × 10^−3^	2.31 × 10^−4^	1.62 × 10^−2^	4.28 × 10^−4^	2.97 × 10^−2^	0.05
Ginger	4.60 × 10^−3^	3.63 × 10^−4^	1.47 × 10^−2^	3.12 × 10^−4^	8.40 × 10^−2^	0.09
White pepper	7.00 × 10^−3^	4.16 × 10^−4^	2.30 × 10^−2^	8.25 × 10^−4^	3.77 × 10^−2^	0.07
Nutmeg	8.67 × 10^−3^	1.70 × 10^−4^	0.85 × 10^−2^	2.75 × 10^−4^	1.63 × 10^−2^	0.04
Negro pepper	9.33 × 10^−3^	3.01 × 10^−4^	1.72 × 10^−2^	4.45 × 10^−4^	4.05 × 10^−2^	0.07
Aniseed	11.33 × 10^−3^	2.02 × 10^−4^	2.08 × 10^−2^	9.72 × 10^−4^	4.06 × 10^−2^	0.07
Rosemary	2.60 × 10^−1^	3.88 × 10^−4^	1.23 × 10^−2^	0.95 × 10^−4^	3.08 × 10^−2^	0.30
Turmeric powder	8.90 × 10^−3^	1.94 × 10^−4^	2.00 × 10^−2^	3.07 × 10^−4^	2.40 × 10^−2^	0.05
Garlic	5.50 × 10^−1^	3.59 × 10^−5^	26.00 × 10^−2^	0.18 × 10^−4^	3.25 × 10^−2^	0.84

## Data Availability

Data are available upon request.

## Supporting Information

The supplementary materials consist of Appendix 1a, which contains data on estimated concentrations (mg/kg) ± mean standard deviation of the heavy metals in the spices. Appendix 1b contains estimated mean concentrations of the heavy metals in each spice from the three markets, and Appendix 2 contains the calibration curves for the elemental analyses.
