# Supplementary material for: Trace and Heavy Metals in Locally and Imported Spices Sold on Markets in Accra Metropolis, Ghana
Source: ScientificWorldJournal. 2024 Oct 24;2024:3168279. doi: 10.1155/2024/3168279 (PMC11527527; doi:10.1155/2024/3168279)
Supplement: Supplementary Materials — Additional supporting information can be found online in the Supporting Information section. [file 3168279.f1.docx]

**APPENDICES**

**APPENDIX 1A - ESTIMATED CONCENTRATIONS (mg/kg) ± MEAN STANDARD DEVIATION OF THE HEAVY METALS IN**

**THE SPICES.**

| **ORIGIN** | **COMMODITY** | **MARKET** | **ID** | **BLOCK** | **Fe(mg/kg) ± SD** | | **As(mg/kg) ± SD** | | **Zn(mg/kg) ± SD** | | **Cd(mg/kg) ± SD** | | **Pb(mg/kg) ± SD** | |
| --- | --- | --- | --- | --- | --- | --- | --- | --- | --- | --- | --- | --- | --- | --- |
| GHANA | GINGER | MADINA | S1 | M1 | 1.613 | 0.346 | 0.087 | 0.019 | 0.625 | 0.043 | 0.156 | 0.030 | 1.892 | 0.957 |
| GHANA | GINGER | MADINA | S2 | M1 | 1.629 |  | 0.056 |  | 0.589 |  | 0.054 |  | 3.583 |  |
| GHANA | GINGER | MADINA | S3 | M1 | 1.594 |  | 0.123 |  | 0.711 |  | 0.088 |  | 0.89 |  |
| GHANA | GINGER | KANESHIE | S1 | M2 | 0.558 |  | 0.099 |  | 0.668 |  | 0.089 |  | 2.214 |  |
| GHANA | GINGER | KANESHIE | S2 | M2 | 0.893 |  | 0.068 |  | 0.689 |  | 0.062 |  | 2.986 |  |
| GHANA | GINGER | KANESHIE | S3 | M2 | 1.187 |  | 0.076 |  | 0.692 |  | 0.082 |  | 0.998 |  |
| GHANA | GINGER | MAKOLA | S1 | M3 | 1.466 |  | 0.072 |  | 0.597 |  | 0.122 |  | 2.108 |  |
| GHANA | GINGER | MAKOLA | S2 | M3 | 1.425 |  | 0.071 |  | 0.628 |  | 0.068 |  | 3.056 |  |
| GHANA | GINGER | MAKOLA | S3 | M3 | 1.429 |  | 0.092 |  | 0.688 |  | 0.072 |  | 0.825 |  |
| GHANA | NEGRO PEPPER | MADINA | S1 | M1 | 0.758 | 1.323 | 0.195 | 0.029 | 0.625 | 0.198 | 0.145 | 0.034 | 1.025 | 0.084 |
| GHANA | NEGRO PEPPER | MADINA | S2 | M1 | 1.346 |  | 0.216 |  | 0.596 |  | 0.048 |  | 0.856 |  |
| GHANA | NEGRO PEPPER | MADINA | S3 | M1 | 1.425 |  | 0.115 |  | 0.225 |  | 0.125 |  | 0.912 |  |

| GHANA | NEGRO PEPPER | KANESHIE | S1 | M2 | 2.223 |  | 0.166 |  | 0.772 |  | 0.117 |  | 1.118 |  |
| --- | --- | --- | --- | --- | --- | --- | --- | --- | --- | --- | --- | --- | --- | --- |
| GHANA | NEGRO PEPPER | KANESHIE | S2 | M2 | 1.461 |  | 0.188 |  | 0.697 |  | 0.055 |  | 0.954 |  |
| GHANA | NEGRO PEPPER | KANESHIE | S3 | M2 | 2.356 |  | 0.163 |  | 0.302 |  | 0.121 |  | 0.958 |  |
| GHANA | NEGRO PEPPER | MAKOLA | S1 | M3 | 5.242 |  | 0.149 |  | 0.669 |  | 0.108 |  | 0.956 |  |
| GHANA | NEGRO PEPPER | MAKOLA | S2 | M3 | 2.128 |  | 0.174 |  | 0.708 |  | 0.072 |  | 0.887 |  |
| GHANA | NEGRO PEPPER | MAKOLA | S3 | M3 | 0.414 |  | 0.142 |  | 0.285 |  | 0.136 |  | 1.089 |  |
| GHANA | GREEN PEPPER | MADINA | S1 | M1 | 2.568 | 0.892 | 0.089 | 0.052 | 0.085 | 0.031 | 0.126 | 0.035 | 0 | 0.110 |
| GHANA | GREEN PEPPER | MADINA | S2 | M1 | 2.447 |  | 0 |  | 0.075 |  | 0.085 |  | 0.258 |  |
| GHANA | GREEN PEPPER | MADINA | S3 | M1 | 2.451 |  | 0 |  | 0.142 |  | 0.045 |  | 0.112 |  |
| GHANA | GREEN PEPPER | KANESHIE | S1 | M2 | 0.894 |  | 0.096 |  | 0.069 |  | 0.096 |  | 0 |  |
| GHANA | GREEN PEPPER | KANESHIE | S2 | M2 | 0.775 |  | 0 |  | 0.056 |  | 0.094 |  | 0 |  |
| GHANA | GREEN PEPPER | KANESHIE | S3 | M2 | 1.025 |  | 0.142 |  | 0.154 |  | 0.052 |  | 0.129 |  |

| GHANA | GREEN PEPPER | MAKOLA | S1 | M3 | 3.569 |  | 0.075 |  | 0.088 |  | 0.128 |  | 0 |  |
| --- | --- | --- | --- | --- | --- | --- | --- | --- | --- | --- | --- | --- | --- | --- |
| GHANA | GREEN PEPPER | MAKOLA | S2 | M3 | 2.447 |  | 0.082 |  | 0.096 |  | 0.105 |  | 0.305 |  |
| GHANA | GREEN PEPPER | MAKOLA | S3 | M3 | 1.687 |  | 0.128 |  | 0.098 |  | 0.022 |  | 0.104 |  |
| GHANA | WHITE PEPPER | MADINA | S1 | M1 | 5.364 | 1.401 | 0.128 | 0.011 | 0.895 | 0.106 | 0.182 | 0.045 | 0.928 | 0.074 |
| GHANA | WHITE PEPPER | MADINA | S2 | M1 | 4.987 |  | 0.115 |  | 0.589 |  | 0.074 |  | 1.025 |  |
| GHANA | WHITE PEPPER | MADINA | S3 | M1 | 5.814 |  | 0.127 |  | 0.722 |  | 0.216 |  | 0.844 |  |
| GHANA | WHITE PEPPER | KANESHIE | S1 | M2 | 2.457 |  | 0.137 |  | 0.759 |  | 0.145 |  | 0.852 |  |
| GHANA | WHITE PEPPER | KANESHIE | S2 | M2 | 2.126 |  | 0.108 |  | 0.602 |  | 0.089 |  | 1.001 |  |
| GHANA | WHITE PEPPER | KANESHIE | S3 | M2 | 3.025 |  | 0.133 |  | 0.746 |  | 0.118 |  | 0.881 |  |
| GHANA | WHITE PEPPER | MAKOLA | S1 | M3 | 2.445 |  | 0.131 |  | 0.912 |  | 0.148 |  | 0.845 |  |
| GHANA | WHITE PEPPER | MAKOLA | S2 | M3 | 2.845 |  | 0.142 |  | 0.711 |  | 0.092 |  | 0.968 |  |
| GHANA | WHITE PEPPER | MAKOLA | S3 | M3 | 2.124 |  | 0.109 |  | 0.802 |  | 0.174 |  | 0.801 |  |

| Indian | Garlic | MADINA | S1 | M1 | 0.128 | 0.042 | 13.89 | 4.353 | 0.867 | 0.143 | 0.245 | 0.054 | 0.758 | 0.103 |
| --- | --- | --- | --- | --- | --- | --- | --- | --- | --- | --- | --- | --- | --- | --- |
| Indian | Garlic | MADINA | S2 | M1 | 0.088 |  | 12.958 |  | 0.523 |  | 0.148 |  | 0.958 |  |
| Indian | Garlic | MADINA | S3 | M1 | 0.022 |  | 14.012 |  | 0.624 |  | 0.089 |  | 0.622 |  |
| Indian | Garlic | KANESHIE | S1 | M2 | 0.122 |  | 12.084 |  | 0.824 |  | 0.231 |  | 0.882 |  |
| Indian | Garlic | KANESHIE | S2 | M2 | 0.061 |  | 13.071 |  | 0.586 |  | 0.14 |  | 0.855 |  |
| Indian | Garlic | KANESHIE | S3 | M2 | 0.032 |  | 11.089 |  | 0.589 |  | 0.095 |  | 0.689 |  |
| Morocco | Garlic | MAKOLA | S1 | M3 | 0.134 |  | 3.456 |  | 0.812 |  | 0.189 |  | 0.689 |  |
| Morocco | Garlic | MAKOLA | S2 | M3 | 0.068 |  | 3.897 |  | 0.569 |  | 0.159 |  | 0.842 |  |
| Morocco | Garlic | MAKOLA | S3 | M3 | 0.028 |  | 4.021 |  | 0.428 |  | 0.105 |  | 0.728 |  |
| China | ROSMARY | MADINA | S1 | M1 | 0.286 | 0.131 | 4.258 | 2.780 | 0.789 | 0.101 | 0.028 | 0.035 | 0.458 | 0.177 |
| China | ROSMARY | MADINA | S2 | M1 | 0.621 |  | 8.751 |  | 0.628 |  | 0.078 |  | 0.985 |  |
| China | ROSMARY | MADINA | S3 | M1 | 0.447 |  | 2.586 |  | 0.664 |  | 0.106 |  | 0.768 |  |
| China | ROSMARY | KANESHIE | S1 | M2 | 0.302 |  | 3.248 |  | 0.766 |  | 0.034 |  | 0.559 |  |
| China | ROSMARY | KANESHIE | S2 | M2 | 0.241 |  | 10.472 |  | 0.529 |  | 0.102 |  | 0.892 |  |
| China | ROSMARY | KANESHIE | S3 | M2 | 0.522 |  | 2.446 |  | 0.608 |  | 0.114 |  | 0.845 |  |
| China | ROSMARY | MAKOLA | S1 | M3 | 0.225 |  | 3.894 |  | 0.712 |  | 0.019 |  | 0.528 |  |
| China | ROSMARY | MAKOLA | S2 | M3 | 0.428 |  | 4.879 |  | 0.887 |  | 0.098 |  | 0.721 |  |
| China | ROSMARY | MAKOLA | S3 | M3 | 0.497 |  | 1.998 |  | 0.704 |  | 0.085 |  | 0.911 |  |
| Burkina Faso | ANI SEED | MADINA | S1 | M1 | 3.456 | 0.536 | 0.257 | 0.027 | 0.368 | 0.077 | 0.084 | 0.118 | 1.258 | 0.185 |

| Burkina Faso | ANI SEED | MADINA | S2 | M1 | 5.218 |  | 0.165 |  | 0.336 |  | 0.453 |  | 0.968 |  |
| --- | --- | --- | --- | --- | --- | --- | --- | --- | --- | --- | --- | --- | --- | --- |
| Mali | ANI SEED | MADINA | S3 | M1 | 4.326 |  | 0.186 |  | 0.421 |  | 0.102 |  | 0.966 |  |
| Mali | ANI SEED | KANESHIE | S1 | M2 | 3.429 |  | 0.228 |  | 0.342 |  | 0.08 |  | 1.114 |  |
| Mali | ANI SEED | KANESHIE | S2 | M2 | 4.023 |  | 0.225 |  | 0.219 |  | 0.042 |  | 0.655 |  |
| Mali | ANI SEED | KANESHIE | S3 | M2 | 4.227 |  | 0.176 |  | 0.503 |  | 0.114 |  | 0.987 |  |
| Mali | ANI SEED | MAKOLA | S1 | M3 | 3.554 |  | 0.211 |  | 0.392 |  | 0.097 |  | 0.985 |  |
| Mali | ANI SEED | MAKOLA | S2 | M3 | 4.116 |  | 0.189 |  | 0.287 |  | 0.058 |  | 0.698 |  |
| Mali | ANI SEED | MAKOLA | S3 | M3 | 4.421 |  | 0.201 |  | 0.398 |  | 0.096 |  | 1.148 |  |
| France | NUTMEG | MADINA | S1 | M1 | 0.825 | 0.209 | 0.245 | 0.088 | 0.162 | 0.135 | 0.027 | 0.017 | 0.148 | 0.230 |
| France | NUTMEG | MADINA | S2 | M1 | 1.212 |  | 0.198 |  | 0.195 |  | 0.048 |  | 0.685 |  |
| Italy | NUTMEG | MADINA | S3 | M1 | 1.358 |  | 0 |  | 0.589 |  | 0.061 |  | 0.258 |  |
| Italy | NUTMEG | KANESHIE | S1 | M2 | 0.921 |  | 0.201 |  | 0.205 |  | 0.034 |  | 0.182 |  |
| Italy | NUTMEG | KANESHIE | S2 | M2 | 1.322 |  | 0 |  | 0.221 |  | 0.079 |  | 0.771 |  |
| France | NUTMEG | KANESHIE | S3 | M2 | 1.402 |  | 0.145 |  | 0.428 |  | 0.052 |  | 0.324 |  |
| France | NUTMEG | MAKOLA | S1 | M3 | 0.899 |  | 0.195 |  | 0.305 |  | 0.031 |  | 0.204 |  |
| France | NUTMEG | MAKOLA | S2 | M3 | 1.148 |  | 0.234 |  | 0.235 |  | 0.057 |  | 0.662 |  |
| Spain | NUTMEG | MAKOLA | S3 | M3 | 1.332 |  | 0.187 |  | 0.417 |  | 0.072 |  | 0.289 |  |
| Indian | TRUMERIC POWDER | MADINA | S1 | M1 | 1.238 | 0.496 | 5.284 | 1.610 | 0.402 | 0.169 | 0.075 | 0.059 | 0.528 | 0.129 |

| Indian | TRUMERIC POWDER | MADINA | S2 | M1 | 1.068 |  | 8.257 |  | 0.208 |  | 0.079 |  | 0.623 |  |
| --- | --- | --- | --- | --- | --- | --- | --- | --- | --- | --- | --- | --- | --- | --- |
| Indian | TRUMERIC POWDER | MADINA | S3 | M1 | 2.258 |  | 2.985 |  | 0.215 |  | 0.196 |  | 0.802 |  |
| Indian | TRUMERIC POWDER | KANESHIE | S1 | M2 | 0.988 |  | 6.025 |  | 0.375 |  | 0.108 |  | 0.611 |  |
| Indian | TRUMERIC POWDER | KANESHIE | S2 | M2 | 0.687 |  | 6.259 |  | 0.306 |  | 0.062 |  | 0.745 |  |
| Indian | TRUMERIC POWDER | KANESHIE | S3 | M2 | 1.987 |  | 3.065 |  | 0.311 |  | 0.251 |  | 0.429 |  |
| Indian | TRUMERIC POWDER | MAKOLA | S1 | M3 | 1.428 |  | 4.986 |  | 0.524 |  | 0.086 |  | 0.489 |  |
| Indian | TRUMERIC POWDER | MAKOLA | S2 | M3 | 0.789 |  | 3.894 |  | 0.705 |  | 0.125 |  | 0.388 |  |
| Indian | TRUMERIC POWDER | MAKOLA | S3 | M3 | 1.187 |  | 4.002 |  | 0.114 |  | 0.099 |  | 0.611 |  |
| Malaysia | BLACK PEPPER | MADINA | S1 | M1 | 1.045 | 1.015 | 0.148 | 0.032 | 0.639 | 0.238 | 0.041 | 0.037 | 0.957 | 0.321 |
| Malaysia | BLACK PEPPER | MADINA | S2 | M1 | 3.589 |  | 0.069 |  | 0.428 |  | 0.152 |  | 0.825 |  |
| Malaysia | BLACK PEPPER | MADINA | S3 | M1 | 0.658 |  | 0.097 |  | 0.074 |  | 0.089 |  | 0.426 |  |
| Malaysia | BLACK PEPPER | KANESHIE | S1 | M2 | 1.548 |  | 0.077 |  | 0.611 |  | 0.053 |  | 1.024 |  |
| Indian | BLACK PEPPER | KANESHIE | S2 | M2 | 3.011 |  | 0.051 |  | 0.523 |  | 0.128 |  | 0.925 |  |
| Malaysia | BLACK PEPPER | KANESHIE | S3 | M2 | 0.958 |  | 0.085 |  | 0.098 |  | 0.125 |  | 0.112 |  |
| Indian | BLACK PEPPER | MAKOLA | S1 | M3 | 1.247 |  | 0.029 |  | 0.702 |  | 0.078 |  | 1.057 |  |
| Malaysia | BLACK PEPPER | MAKOLA | S2 | M3 | 2.897 |  | 0.058 |  | 0.552 |  | 0.136 |  | 0.771 |  |
| Malaysia | BLACK PEPPER | MAKOLA | S3 | M3 | 1.124 |  | 0.061 |  | 0.113 |  | 0.069 |  | 0.325 |  |

**APPENDIX 1B**

**Estimated mean concentrations of the heavy metals in each spice from the three markets**

|  |  | **AS** |  |  | **Zn** |  |  | **Cd** |  |
| --- | --- | --- | --- | --- | --- | --- | --- | --- | --- |
|  | **MADINA** | **KANESHIE** | **MAKOLA** | **MADINA** | **KANESHIE** | **MAKOLA** | **MADINA** | **KANESHIE** | **MAKOLA** |
| *Green Pepper* | 0.030 | 0.079 | 0.095 | 0.101 | 0.093 | 0.094 | 0.085 | 0.081 | 0.085 |
| *Black Pepper* | 0.105 | 0.071 | 0.049 | 0.380 | 0.411 | 0.456 | 0.094 | 0.102 | 0.094 |
| *Ginger* | 0.089 | 0.081 | 0.078 | 0.642 | 0.683 | 0.638 | 0.099 | 0.078 | 0.087 |
| *White Pepper* | 0.123 | 0.126 | 0.127 | 0.735 | 0.702 | 0.808 | 0.093 | 0.117 | 0.138 |
| *Nutmeg* | 0.148 | 0.115 | 0.205 | 0.315 | 0.285 | 0.319 | 0.045 | 0.055 | 0.053 |
| *Negro Pepper* | 0.175 | 0.172 | 0.155 | 0.482 | 0.590 | 0.554 | 0.106 | 0.098 | 0.105 |
| *Ani Seed* | 0.203 | 0.210 | 0.200 | 0.375 | 0.355 | 0.359 | 0.213 | 0.079 | 0.084 |
| *Rosemary* | 5.198 | 5.389 | 3.590 | 0.694 | 0.634 | 0.768 | 0.071 | 0.083 | 0.067 |
| *Turmeric Powder* | 5.509 | 5.116 | 4.294 | 0.275 | 0.331 | 0.448 | 0.117 | 0.140 | 0.103 |
| *Garlic* | 13.620 | 12.081 | 3.791 | 0.671 | 0.666 | 0.603 | 0.161 | 0.155 | 0.151 |

|  | **Fe** |  |  | **Pb** | | |
| --- | --- | --- | --- | --- | --- | --- |
|  | **MADINA** | **KANESHIE** | **MAKOLA** | **MADINA** | **KANESHIE** | **MAKOLA** |
| *Green Pepper* | 2.489 | 0.898 | 2.568 | 0.123 | 0.043 | 0.136 |
| *Black Pepper* | 1.764 | 1.839 | 1.756 | 0.736 | 0.687 | 0.718 |
| *Ginger* | 1.612 | 1.369 | 1.440 | 2.122 | 2.066 | 1.996 |
| *White Pepper* | 5.388 | 2.536 | 2.471 | 0.994 | 0.911 | 0.871 |
| *Nutmeg* | 1.132 | 1.215 | 1.126 | 0.364 | 0.426 | 0.385 |
| *Negro Pepper* | 1.176 | 2.013 | 2.595 | 0.931 | 1.010 | 0.977 |
| *Ani Seed* | 4.333 | 3.893 | 4.030 | 1.064 | 0.919 | 0.944 |
| *Rosemary* | 0.451 | 0.355 | 0.383 | 0.737 | 0.765 | 0.720 |
| *Turmeric Powder* | 1.521 | 1.221 | 1.135 | 0.651 | 0.595 | 0.496 |
| *Garlic* | 0.079 | 0.072 | 0.077 | 0.779 | 0.809 | 0.753 |

# APPENDIX 2: CALIBRATION CURVES FOR THE ELEMENTAL ANALYSES APPENDIX 2A

y = 0.0062x + 0.0195

R² = 0.9908

0

0.05

0.1

0.15

0.2

0.25

0.3

0.35

0

10

20

30

40

50

60

**Absorbance**

**Concentration (ppb)**

**Calibration Curve of Iron (Fe)**

# APPENDIX 2B

y = 6.2192x + 18.69

R² = 0.9903

0

50

100

150

200

250

300

350

0

10

20

30

40

50

60

**Absorbance**

**Concentration (ppb)**

**Calibration Curve of**

**Cadmium (Cd)**

# APPENDIX 2C

y = 149.92x

-

186.62

R² = 0.9932

-1000

0

1000

2000

3000

4000

5000

6000

7000

8000

0

10

20

30

40

50

60

**Absorbance**

**Concentration (ppb)**

**Calibration Curve of**

**Arsenic (As)**

# APPENDIX 2D

y = 34.535x + 65.4

R² = 0.9908

0

200

400

600

800

1000

1200

1400

1600

1800

2000

0

10

20

30

40

50

60

**Absorbance**

**Concentration (ppb)**

**Calibration Curve of**

**lead (Pb)**

# APPENDIX 2E

y = 6.6008x + 5.4855

R² = 0.9977

0

50

100

150

200

250

300

350

400

0

10

20

30

40

50

60

**Absorbance**

**Concentration (ppb)**

**Calibration Curve of**

**Zinc (Zn)**
